# *In vitro* percutaneous penetration test overview

**DOI:** 10.3389/fphar.2023.1102433

**Published:** 2023-06-14

**Authors:** Sheeva Shahinfar, Howard Maibach

**Affiliations:** ^1^ Texas A&M University School of Medicine, Bryan, TX, United States; ^2^ UCSF Dermatology, San Francisco, CA, United States

**Keywords:** percutaneous penetration, *in vitro* vs. *in vivo* skin, percutaneous absorption, topical drugs, transdermal drugs, dermatologic drugs

## Abstract

Skin is a detailed, organized, and intricate niche in the human body. Topical and transdermal drugs are unique, in that their absorption is quite different from other routes of administration (oral, intramuscular, intravenous, etc.,.). A robust amount of research is required to approve the use of a drug—*in vivo*, *in vitro*, and *ex vivo* studies collectively help manufacturers and government agencies with approval of various compounds. Use of human and animal studies poses ethical and financial concerns, making samples difficult to use. *In vitro* and *ex vivo* methods have improved over the past several decades—results show relevance when compared to *in vivo* methods. The history of testing is discussed, followed by a detailed account of known complexities of skin and the current state of percutaneous penetration.

## Introduction

Skin is a highly organized, intricate niche in the human body that, due to its complexity, exhibits a unique nature. Over recent decades, advances have been made in understanding percutaneous absorption of topical and transdermal drugs. Topical drugs include semi-solid creams, gels, ointments, and sprays applied directly to the skin. Transdermal drugs are intended, not for local delivery, but for systemic effects. Examples of transdermal drugs include scopolamine (used for motion sickness), nicotine (used for smoking cessation), and nitroglycerine (used for its antianginal effects). Most of the data presented here will encompass topical drugs, as their main intent was to provide relief for dermatologic conditions, rather than systemic conditions. Drugs applied to skin have a major advantage over oral, intravenous, and intramuscular drugs, namely, being able to bypass hepatic metabolism, not undergoing the sensitive GI tract, potential for long-term drug delivery, and increased patient compliance. Approval of topical drugs requires robust research. It is often challenging to obtain results with human and animal studies due to difficulty obtaining samples/subjects, high cost, and ethical concerns. Moreover, *in vivo* studies do not always allow us to account for mass balance. Thus, recent advances have been made in our use and understanding of *in vitro* and *ex vivo* methods for evaluation of topical drugs.^†^

We trace the evaluation of the early innovations of Burch and Windsor, and Tregear, to current usage of the *in vitro* permeation test—describing subsequent insights into performance and interpretation—as well as defining data gaps where eventual solutions will be proposed and closer approximation of *in vivo* human data. Here, we discuss the history of skin evaluation, current knowledge on permeability of the skin, and future advances that must be made.

## Methods

We compiled our research using PubMed’s search engine tool. The following terms were inputted: “*in vitro in vivo* skin”, “skin barrier”, “structure of skin”, “skin permeability”, “stratum corneum permeability”, “percutaneous absorption”. A thorough review of the literature from 1960 onwards was completed and referenced. The UCSF dermatopharmacology-dermatotoxicology library was hand searched.

## Results

**FIGURE 1:**
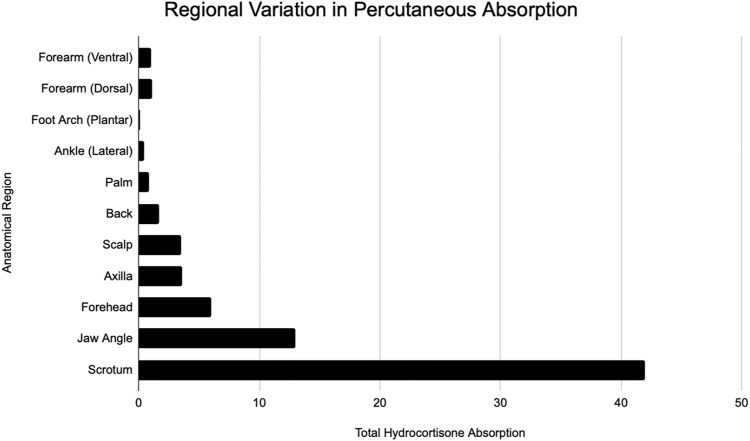
Modified from Feldman and Maibach (Feldmann and Maibach, 1967). Regional variation in percutaneous absorption of hydrocortisone.

The skin is a highly organized organ that can be described and depicted in various ways. The major advantage of using excised skin *in vitro* as opposed to intact humans is the easier capability of measuring the penetrant (Tregear, 1966). Of the many *in vivo* studies that have been done, Bucks et al. is one that compared the percutaneous absorption of four steroids - hydrocortisone, estradiol, testosterone, and progesterone. Tables 1 and 2 show the distribution of these compounds on the skin following a single dose under occluded and protected conditions (Bucks et al., 1988). Feldmann and Maibach performed groundbreaking *in vivo* studies, where they determined the effect that anatomical sites have on percutaneous absorption, a concept referred to as regional variation (Figure 1). (Feldmann and Maibach, 1967) Table 3 shows Guy and Maibach’s results, which furthered Feldmann and Maibach’s study and expanded it to be used as a clinical guideline for prescribing compounds to be applied on different anatomical sites (Bronaugh et al., 1985). Throughout the next decades, the complexity of percutaneous absorption continued to be discovered—Law et al. have most recently described the twenty most clinically pertinent factors when evaluating the penetration and efficacy of a topical drug. Their results are depicted in Table 4 (Law et al., 2020).

**TABLE 1:** Modified from Bucks et al. (Bucks et al., 1988) Percent disposition of topically applied 14C-labeled steroids after a single dose under occluded conditions. HTC = Hilltop chamber. ND = not determined.

Steroid	Absorbed	1st HTC	1st Wash	2nd HTC	2nd Wash	Total
Hydrocortisone	4.0 ± 2.4	28 ± 5.6	36 ± 3.0	ND	ND	68 ± 3.9
Estradiol	27 ± 6.4	41 ± 10	18 ± 7.2	0.5 ± 0.3	ND	87 ± 13
Testosterone	46 ± 15	41​​ ± 8.4	3.0 ± 4.1	0.3 ± 0.2	ND	90 ± 8.4
Progesterone	33 ± 8.9	46 ± 10	1.2 ± 0.8	0.07 ± 0.02	0.01 ± 0.0	80 ± 5.5

**TABLE 2:** Modified from Bucks et al. (Bucks et al., 1988) Percent disposition of topically applied 14C-labeled steroids after a single dose under protected (“non-occlusive”) conditions. HTC = Hilltop chamber. ND = not determined. SC “strips” included 14C-radiolabel found in 10 tape strippings of stratum corneum removed after final wash.

Steroid	Absorbed	1st HTC	1st Wash	2nd HTC	2nd Wash	SC “strips”	Total
Hydrocortisone	4.4 ± 1.7	27 ± 11	51 ± 18	3.2 ± 1.7	2.7 ± 1.3	2.5 ± 1.1	89 ± 5.6
Estradiol	3.4 ± 1.2	38 ± 13	58 ± 12	0.7 ± 0.4	0.3 ± 0.4	0.1 ± 0.1	100 ± 0.9
Testosterone	18 ± 8.6	46​​ ± 7.5	30 ± 15	1.4 ± 0.4	0.1 ± 0.08	ND	96 ± 2.0
Progesterone	13 ± 6.3	54 ± 7.7	27 ± 8.7	1.2 ± 0.6	0.3 ± 0.4	ND	96 ± 3.4

**TABLE 3:** Modified from Guy and Maibach (Bronaugh et al., 1985). Penetration indices for five anatomical sites (genitals, arms, legs, trunk, head) with relation to surface areas. Used when considering systemic availability relativity to body exposure areas.

	Adults	Area (cm^2^)	Neonates	Area (cm^2^)
Anatomical Region	Body area (%)		Body area (%)	
Genitals	1	190	1	19
Arms	18	3,420	19	365
Legs	36	6,840	30	576
Trunk	36	6,840	31	595
Head	9	1,710	19	365
Total		19,000		1,920

**TABLE 4:** Modified from Law and Maibach (Law et al., 2020). Twenty clinically pertinent factors of percutaneous absorption.

1	Relevant physico-chemical properties (particle size/molecular weight, lipophilicity, pH, pK_a_, partition coefficient)
2	Vehicle/formulation
3	Conditions of drug exposure (dose, duration, surface area, frequency of exposure)
4	Skin appendages (glands, hair follicles) as sub-anatomical pathways
5	Skin application sites (regional variation in penetration)
6	Population variability (prematurity, infants, elderly)
7	Skin surface conditions (hydration, pH, temperature)
8	Skin health and integrity (trauma, skin disease)
9	Substantivity and binding to different skin components
10	Systemic distribution and systemic toxicity
11	Exfoliation
12	Washing-off and washing-in
13	Rubbing/massaging
14	Transfer to others (human to human or hard surface to human)
15	Volatility
16	Metabolic biotransformation and cutaneous metabolism
17	Photochemical transformation and photosensitivity
18	Excretion pharmacokinetics
19	Lateral spread
20	Chemical method of determining percutaneous absorption

**FIGURE 2:**
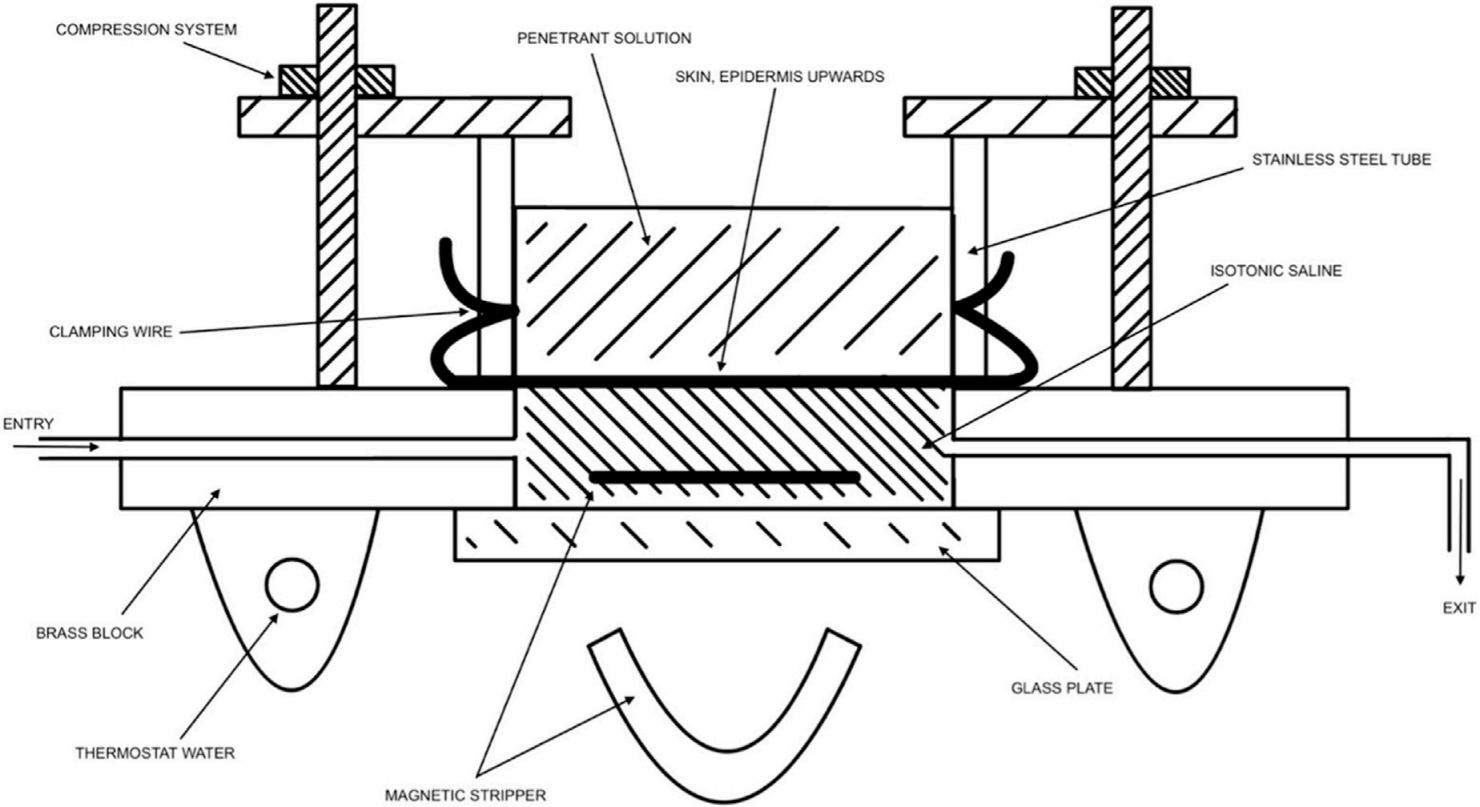
Modified from Tregear (Tregear, 1966). Visual representation of diffusion cells being used for measurement of penetration by isotope-labeled chemicals through the skin.

*In vitro* and *ex vivo* studies attempt to understand the effect of substances without the use of a live human subject (*in vivo*). *In vitro* studies consist of an artificial cell system, created in a lab, to emulate the *in vivo* environment. *Ex vivo* studies consist of human or animal tissue taken as a harvest from the subject, also attempting to emulate the *in vivo* environment. *Ex vivo* studies most popularly use a standardized diffusion cell. Using such cell, the penetrant can be easily removed from the skin and placed in fluid (e.g., saline). In artificial studies, the collected penetrant has passed through the full dermis, whereas in in vivo studies, the penetrant has most likely been collected in urine, blood, feces, and/or expired air. Figure 2 visually depicts an adaptation of Tregear’s set up of excised skin in diffusion cells for *ex vivo* experiments (Tregear, 1966). Bronaugh compared a static diffusion cell to a flow-through system. A comparison of absorption based on his experiments on water, cortisone, and benzoic acid are shown in Table 5 (Bronaugh and Stewart, 1985). Ajjarapu updated this information by comparing data between flow-through and static diffusion cells from different studies. His results showed that flow-through and static diffusion cells indeed often produce similar results with the exception of two variables. Time differences and the make-up of the chemical play an important role in the success of the diffusion system used. He noted that hydrophilic drugs fare better in a static system compared to lipophilic drugs (Ajjarapu and Maibach, 2022).

**TABLE 5:** Modified from Bronaugh et al. (Bronaugh and Stewart, 1985) Comparison of flow-through and static-diffusion cell absorption. Values are listed as the mean ± standard error of the mean of the number of determinations in parentheses. Cortisone and benzoic acid were given with an acetone vehicle. Their absorption is displayed as the percent of the dose absorbed in 24 h. Water was given with a water vehicle and the steady state rate of absorption is expressed in μg/cm^2^⋅h.

Compound	Flow cell	Static cell
Water	4.3 ± 0.4 (5)	4.4 ± 0.2 (5)
Cortisone	8.5 ± 0.9 (5)	6.3 ± 0.8 (8)
Benzoic Acid	45.9 ± 7.6 (5)	48.6 ± 3.8 (6)

**FIGURE 3:**
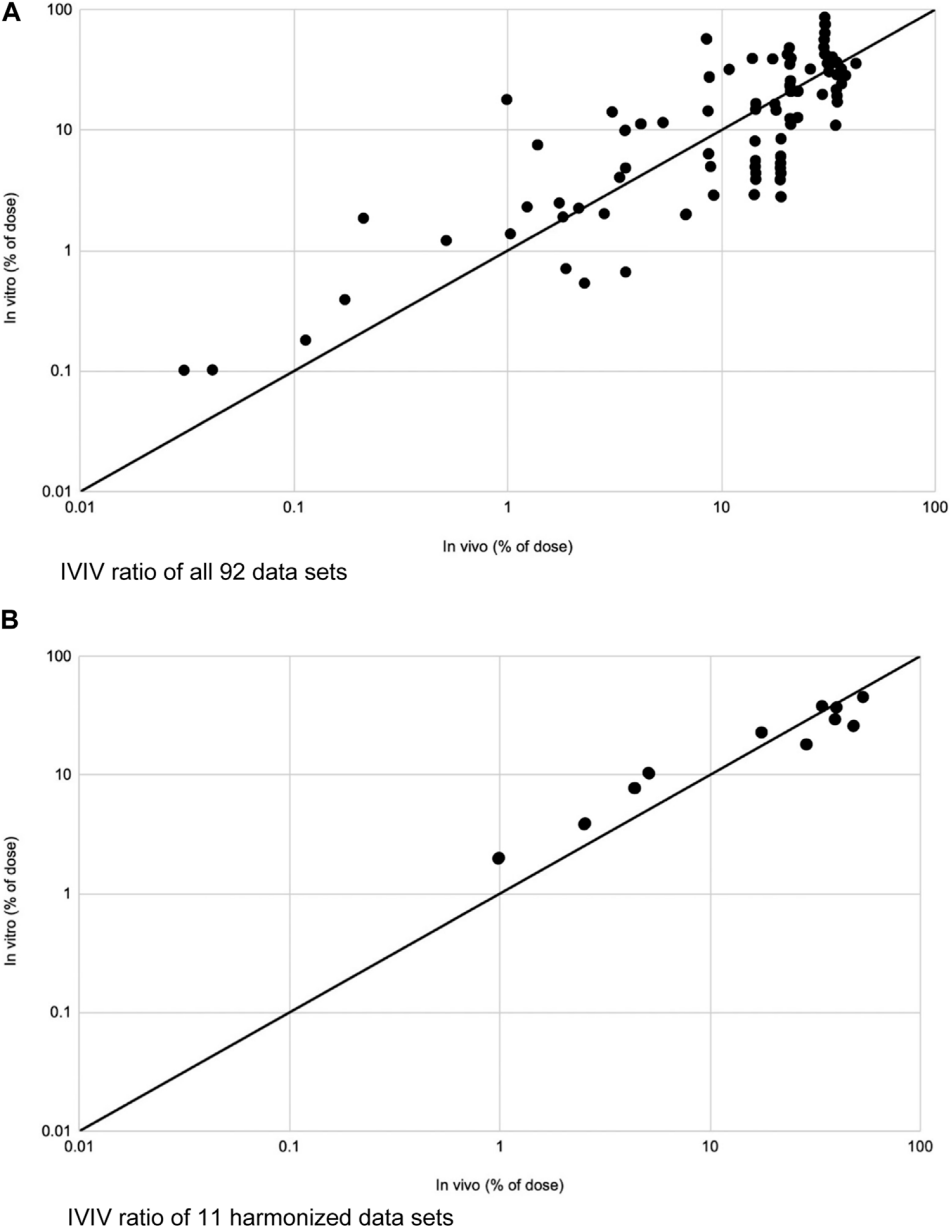
Modified from Lehman et al. (Lehman et al., 2011). **(A)** IVIV ratios of total absorption of 92 data sets plotted on a logarithmic scale. IVIV ratios ranged from 0.18 to 19.7. Mean was 1.6. Solid line depicts an ideal 1:1 IVIV correlation. **(B)** IVIV ratios of total absorption for 11 fully harmonized data sets on logarithmic scale. IVIV ratios ranged from 0.58 to 1.28. Mean was 0.96. Solid line depicts an ideal 1:1 IVIV correlation.

When comparing *in vitro* to *in vivo* studies, Figure 3 depicts IVIV ratios of 92 data sets as well as 11 harmonized data sets. By and large, when data is harmonized and confounding variables are removed, there is a near 1:1 ratio between results seen in in vitro and *in vivo* studies of the same drug.

## Discussion

### History of permeation

Initial recognitions of the power of the skin barrier came from chemical warfares used in World War I. Prior to World War I, there was little known about percutaneous penetration. The research of the United States Army, the American University Experiment Station Laboratories, and Western Reserve University, laid the groundwork of our understanding of the effect of foreign chemicals on skin (Jiang and Maibach, 2018). Their work included our foundational knowledge of percutaneous absorption and the effect of various vehicles used on skin.

Nearly 50 years later, Tregear visually described the apparatus used for *in vitro* experiments (Figure 2). (Tregear, 1966) Tregear’s work, alongside Burch et al., noted that recently dead skin does not affect the permeability of the skin, a major development in our ability to use excised skin in diffusion cells. This was shown experimentally for both water and tri-n-butyl phosphate (Burch and Winsor, 1946; Bronaugh and Stewart, 1984). Thus, excised skin can be used for measuring permeability. Of note, antibiotics are frequently used on excised skin to decrease the chance of bacterial degradation.

### Skin compartments and mass balance

The detailed nature of skin is still not fully understood but advances are made frequently in the field of dermatology. In order to understand the depth of percutaneous absorption, it is necessary to have a foundational understanding of its compartments. Skin is composed of three primary layers—epidermis, dermis, and hypodermis. Epidermis (30–80 μm thick) has several sublayers, each with their own distinctive properties and roles—the stratum corneum (8–20 μm thick), stratum lucidum, stratum granulosum, stratum spinosum, and stratum basale. Stratum corneum will be touched on heavily in this paper, as it is recognized as a primary barrier to absorption. Following the epidermis is the dermal-epidermal junction, which contributes greatly to the dynamic nature and integrity of skin. It is made of four parts—basal keratinocytes and hemidesmosomes, lamina lucida, lamina densa, and sub-basal lamina. The higher layers include proteins such as laminin and nidogen. The lamina dense is composed of type IV collagen and heparin sulfate proteoglycan and the sub-basal lamina densa is composed of anchoring fibrils (Zou and Maibach, 2018). Following the dermal-epidermal junction is the dermis, measuring 1–2 mm. The final layer is the hypodermis, measuring 0.1 to a few centimeters (Agache et al., 2017).

When evaluating percutaneous absorption, the topic of “mass balance” must be considered. Mass balance is the physical concept of accounting for mass composition throughout a system. When a topical drug is applied, we must understand where all components are—are they still on the surface, in the stratum corneum, in the bloodstream, or have they been excreted? Typically, in in vivo techniques, a compound is topically applied, either in a volatile or more complex vehicle. Urinary excretion and/or blood is measured over the next several days. Although helpful, important factors are often not considered—namely, its metabolites may not be evaluated (Bucks et al., 1988).

Bucks et al. modified this technique for more definitive results of hydrocortisone, estradiol, testosterone, and progesterone, by evaluating the effects of both occlusion and protection of skin. In the occlusive experiment, a physical intact chamber is placed directly on the skin, which will often increase percutaneous penetration. In the protective experiment, a plastic Hilltop^®^ chamber was used, which is a chamber not placed directly on the skin, but rather placed above the skin allowing for a level of protection coupled with ventilation. Evaluating for mass balance often is improved by radiochemical tagging, covering the application site to account for sloughing off of particles, washing the skin surface after the dosing period, and sometimes tape stripping the stratum corneum for more complete results (Bucks et al., 1988). Results showed that estradiol, testosterone, and progesterone had much higher absorption rates (measured through higher concentration excreted in urine) under occluded conditions as compared to protected conditions. Tables 1 and 2 compare the distribution of all compounds in both occluded and protected conditions. Hydrocortisone, the least lipophilic drug, was measured at much higher concentrations in the stratum corneum on day 7 compared to the other steroids. This suggests substantivity—specific, strong interactions occurring in the stratum corneum. Other chemicals, similar to hydrocortisone, remain in the stratum corneum for an extended period of time, which may contribute to the toxicity of some drugs when applied topically as opposed to orally.

**FIGURE 4:**
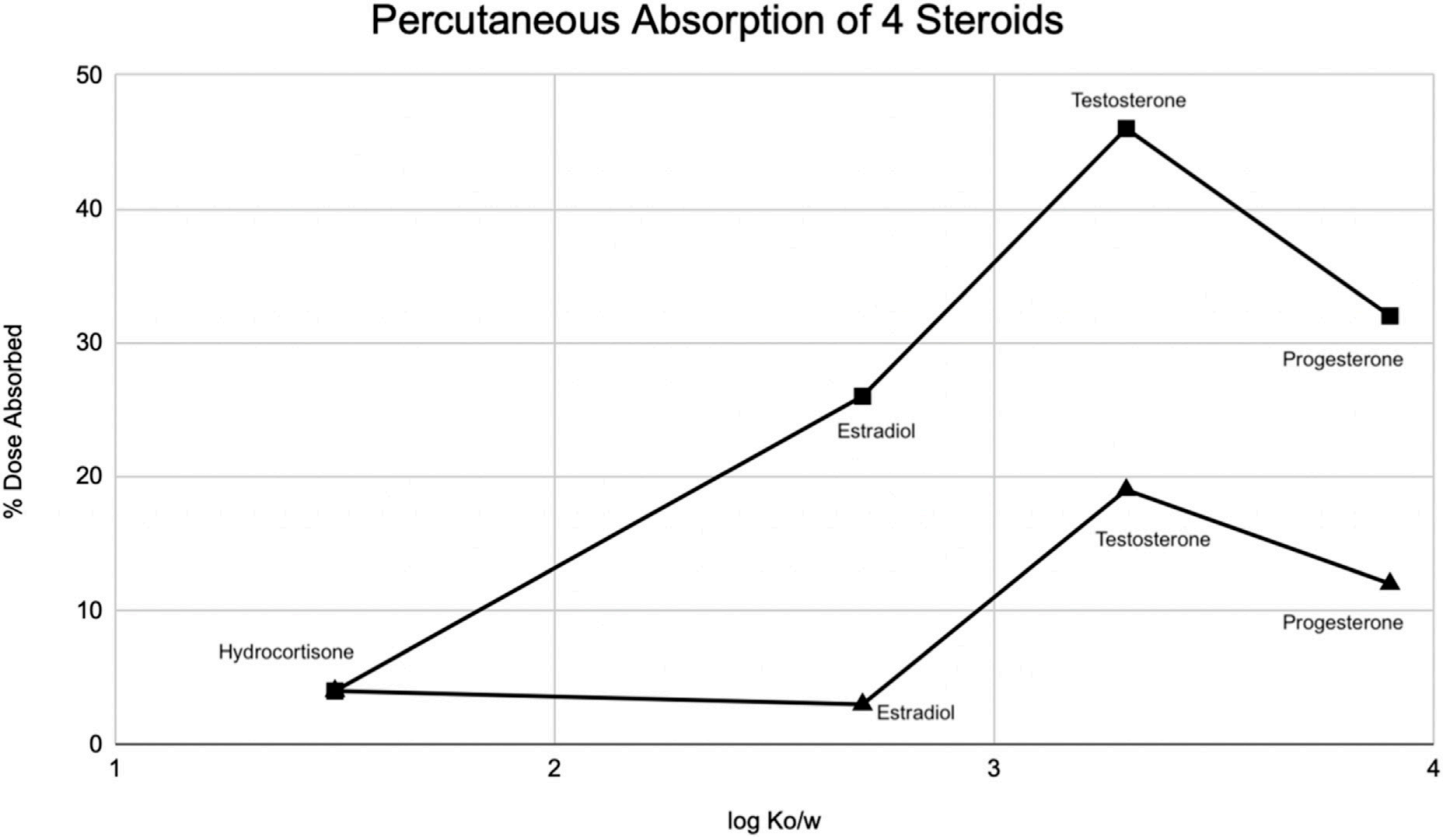
Modified from Bucks et al. (Bucks et al., 1988) Percutaneous absorption of four steroids (hydrocortisone, estradiol, testosterone, progesterone) displayed as a function of octanol/water partition coefficient (K_o/w_) under occluded (squares) and protected (triangle) conditions.

Occlusion significantly increases stratum corneum hydration. The complexity of the skin is seen with this profound finding—if the skin was a simple organ, the hydration from occlusion would equally affect penetration coefficients of all drugs as lipophilicity increases. That is not what we see. Figure 4 shows that progesterone, the most lipophilic compound, has a lower dose absorbed than testosterone (Bucks et al., 1988). Bucks et al. hypothesized that hydrating the stratum corneum decreases the partition coefficient of the compound between the stratum corneum and the epidermis. This, in turn, increases the kinetics of transferring the compound from one layer to the next, and thus, is seen more with more lipophilic compounds. But penetration does not progressively increase with increased lipophilicity. The decrease with progesterone is due to a change in the rate-limiting step of the transfer of compounds from the stratum corneum to the epidermis. Thus, there is a limit to increased absorption with more lipophilic compounds—at one point, we see that a more lipophilic compound actually has a slower relative absorption rate (Bucks et al., 1988). The reason for this stark contrast in percutaneous absorption relative to lipophilicity is not fully understood. It has been observed that a certain amount of the aqueous and lipid solubility aids penetration, but extreme lipid solubility decreases it (Dragicevic and Maibach, 2018).

### Flow-through vs. static systems

The static cell is an easier method of measuring permeation through skin, although it is scientifically preferred to perform experiments via a flow-through system. Ajjarapu et al. notes that the only two factors that may significantly affect the results seen between static and flow-through systems are time differences in sample collection and the chemicals themselves (Ajjarapu and Maibach, 2022). Namely, more water-soluble chemicals may show similar results with both systems, whereas lipophilic chemicals may not.

**FIGURE 5:**
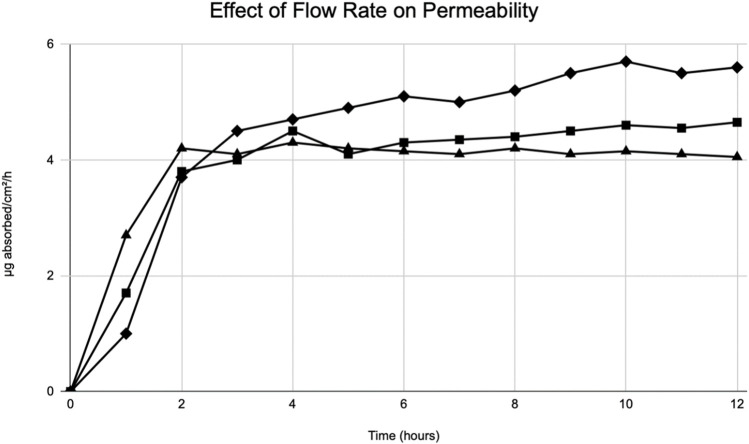
Modified from Bronaugh et al. (Bronaugh and Stewart, 1985) Effect of flow rate on permeability of [^3^H] water. Graphical depiction of the absorption of water through rat skin. Flow rates: 1.0 mL/h (diamonds), 5.0 mL/h (squares), 40 mL/h (triangles).

During World War II, Tregear used static cells to perform *in vitro* experiments on skin. Years later, he laid the foundational work for the flow-through system (Figure 2). (Tregear, 1966) Around the same time, Marzulli’s ground-breaking work set the precedent for the future of flow through *in vitro* skin research (Marzulli, 1962). Bronaugh compared the efficacy of a flow-through system as compared to a static cell. Table 5 details comparison of absorption based on his experiments on water, cortisone, and benzoic acid (Bronaugh and Stewart, 1985). His research showed that a flow rate of at least 5 mL/h is needed for a receptor of volume 0.4 mL to produce accurate results. Moreover, once that minimum flow rate is achieved, increasing the flow rate does not increase absorption (Figure 5). (Bronaugh and Stewart, 1985)

The volume of the receptor is essential to the accuracy of results. The reason for this is that in order to remove the compound that has entered the receptor during a period of time, the volume pumped into the cell must be much larger than the volume of the receptor. Thus, the receptor must have a small volume to compensate for exiting the cell is feasible. Moreover, Bronaugh’s work confirmed that a minimum flow rate must be present in order for proper evaluation to occur. With the minimum flow rate of 5 mL/h for a receptor of volume 0.4 mL, adequate mixing occurs and the absorbed compound is removed from the receptor rapidly, helping decrease the chance for presence of artifact.

However, in cases of using compounds that are poorly water soluble, a different result is seen. Crutcher evaluated the absorption of testosterone and testosterone propionate (two lipophilic compounds) and their results showed that as flow rate increased, absorption also increased. Possible reasons for this could be that they had not reached the minimum rate for a large area of skin they were using or that the compounds they were using were lipophilic, which tend to have increased absorption with increased flow rate. Crutcher pointed out that the increased concentration of compound in the perfusate in their *in vitro* chamber limits the rate of percutaneous penetration (Crutcher and Maibach, 1969). In human skin, concentration in the dermal capillaries is inconsequential, thus, it is important for *in vitro* chambers to replicate physiological conditions by adapting a continuous flow of the perfusate, similar to human skin.

### Skin surrogates

In the quest for finding methods that accurately predict absorption of transdermal compounds, many ethical and financial roadblocks exist. The use of excised human skin may be difficult due to limited availability and cost. Excised animal skin is a method that has been introduced due to its lower cost, however, ethical, and reproducibility concerns may limit their use. Differences in race, age, and sex between animal skins can affect extrapolated results (Neupane et al., 2020).

Due to the various obstacles with using excised human and animal skin as testing models, researchers have used skin surrogates to test compounds. Skin surrogates are broadly separated into three subgroups: artificial human skin models, parallel artificial membrane permeability assays (PAMPAs), and artificial membranes based on polymer/lipid models. The major benefit of using skin surrogates, as compared to animal and human skin, is the consistency in surrogates, leaving minimal room for variations amongst different skin samples.

First, artificial skin models began with a simple design: a de-epidermized dermis was used as the foundation for normal human keratinocytes to grow on (Ponec, 1992). This design was further developed—now, artificial skin samples are recognized in two broad categories: the reconstructed human epidermis model (RHE) and the full thickness model (also known as living skin equivalents, LSE). They differ in that the RHE model allows keratinocytes to grow on the epidermis while the LSE model grows them on both the epidermis and dermis.

Schäfer-Korting et al. evaluated absorption of testosterone across different modalities and showed that RHE models had the highest permeation coefficient, followed by pig skin, bovine udder skin, and human epidermis (Schäfer-Korting et al., 2008). Although RHE had a higher permeation coefficient compared to animal and human skin models, the permeation ranking was similar amongst all samples. Taken together, RHE models can be used as viable alternative methods to human and animal skin for the study of compounds applied as aqueous solutions in in vitro studies. Relevance to human skin *in vivo* remains to be documented.

There has not been a skin model that perfectly resembles human skin. Namely, a commonly used reconstructed skin model has shown to have similar lipid composition of its stratum corneum to human skin, however the higher permeation coefficient is due to its lack of a comparable barrier. Although the lipid composition is the same to human skin, cholesterol was seen as droplets rather than evenly distributed throughout (Tfayli et al., 2014).

The second category of skin surrogates are known as Skin-PAMPAs (skin parallel artificial membrane permeability assay). The design of PAMPAs involves a donor and a receptor compartment, separated by a liquid membrane. The donor compartment includes the compound to be tested. Niacinamide, when applied to both a Franz diffusion cell (*ex vivo*) and to a PAMPA model exhibited a linear correlation between the two models (Zhang et al., 2019). This places PAMPAs as a probable candidate for use as an artificial skin surrogate compared to animal and human skin models. Although more studies must be conducted, PAMPAs may show promise for their efficiency in both time and cost.

The final category of skin surrogates includes artificial skin models based on simple polymers and lipids. An example of this is Strat-M™, made of layers of polyester sulfone. The limitation of this is that it is difficult to control permeation through each layer and measure permeability at each layer, as we do with human skin. Studies have reproduced permeability in Strat-M™ models, although as a general rule, hydrophilic compounds tend to show higher permeability than lipophilic compounds (Neupane et al., 2020). Clinical relevance to human *in vivo* studies has not been documented.

Over recent decades, much work has been done to optimize skin models to be used *in vitro* to mimic human skin. Current models have an epidermis made of robust models of stratum corneum, stratum granulosum, stratum spinosum, and stratum basale (Bouwstra et al., 2021). The most difficult barrier that has been faced are the differences in skin homeostasis and barrier function. Bouwstra et al. postulates that lipid composition, lipid properties, and cornified envelope composition factor into the complexity of the skin barrier that is difficult to reproduce *in vitro* (Bouwstra et al., 2021). In 1988, Ponec et al. showed that exposing a de-epidermized dermis to air significantly improved its lipid composition (Ponec et al., 1988). Mak et al. demonstrated that de-epidermized dermis coupled with 1,25-dihydroxy vitamin D3 helps the barrier resemble that of human skin (Mak et al., 1991). Even still, this model lacked covalently bound lipid lamellae that properly extruded into the regions between cells, which is a foundational part of the skin barrier (Bouwstra et al., 1995; Kennedy et al., 1996). Following this, Boyce and Williams showed that adding palmitic acid, essential fatty acids, and antioxidants increased the synthesis of lipids and improved extrusion (Boyce and Williams, 1993). Ponec et al. discovered that the addition of vitamin C to an air-exposed de-epidermized dermis model and epidermis significantly improved the lipid composition and extrusion process (Ponec et al., 1997). Indeed, as was hoped, the number of triglycerides decreased and the most hydrophilic ceramides increased, improving in lipid composition. This finding was confirmed in permeation studies using corticosterone as well (Pasonen-Seppänen, Suhonen, Kirjavainen, Miettinen, Urtti, Tammi, et al., 2001).

### Regional variation

In addition to the advances that have been made in improving skin surrogates, in order to extrapolate data and translate to compounds used on humans, it is important to evaluate regional variation in human skin. Regional variation is the concept that percutaneous absorption in humans varies in different areas of the body. Consequently, systemic side effects will be different depending on how much of the compound is being absorbed. Feldmann began this quest for understanding the concept of regional variation. Using hydrocortisone, they observed that the scrotum is by far the highest absorbing site tested, followed by regions on the face. The lowest tested absorption site was the forearm (Feldmann and Maibach, 1967). Two decades later, Guy built a table to be referenced by clinicians based off of hydrocortisone and pesticide data for five sites—genitals, arms, legs, trunk, and head (Bronaugh et al., 1985). Table 3 shows these results.

Regional variation has also been tested in animals, which may affect inferences made in humans. Bronaugh showed that rat abdominal skin was more permeable to water, urea, and cortisone than the back skin. This is probably due to the thin skin of the abdomen compared to the back (Bronaugh and Maibach, 1985). As a further example, the rhesus macaque shows regional variation between the forehead and forearm for compounds such as fenitrothion and aminocarb (Moody and Franklin, 1987). Taken together, both animal and human studies documents that variation amongst anatomical sites change the effect of compounds applied to the human skin.

### Metabolism of topical drugs

The metabolism of topical compounds can begin in the skin. Phase I compounds, such as cytochrome P450, and phase II compounds, such as *N*-acetyltransferase, have been seen in skin, although their enzymatic activity, as expected, is significantly lower than their activity in liver (Kazem et al., 2019). Biotransformation effects in the skin can be seen with inactive chemicals becoming active or *vice versa*, or even a lipophilic compound becoming more hydrophilic. These changes can affect the penetration of the compound. As we saw in Buck et al.’s paper on the penetration of four corticosteroids, very lipophilic compounds like progesterone do not absorb well through skin (Bucks et al., 1988). Conversely, with metabolic changes, a very lipophilic compound like butylparaben metabolizes in the skin to *p*-hydroxybenzoic acid, which is more hydrophilic than its pro-drug, increasing its absorption capability. We can also determine the effects of metabolism with the effect of UV rays on compounds—many compounds (psoralens, sulfonamides, tetracyclines) have been identified and known to increase photosensitivity when exposed to UV rays (Law et al., 2020). Once compounds have been absorbed through the skin and entered systemic circulation, the more water-soluble drugs typically can be excreted without further metabolic changes (Law et al., 2020). There is still much to be learned about excretion profiles, metabolism, and clearance rates of topical drugs.

### Protein binding

Several decades ago, little was known about the power of the stratum corneum. Although it was recognized as a main barrier of the skin, its sheer complexity had not yet been elucidated. Today, we know that stratum corneum serves as a primary barrier, limiting compounds from fully penetrating the skin while simultaneously limiting water from exiting the body through skin. This is in part due to the acid mantle, primarily made of phospholipase A2 (Yosipovitch and Maibach, 1996; Zhai et al., 2008). Indeed, after topical application, many compounds are considered “locked into” the stratum corneum (Bronaugh and Maibach, 2005). Furthermore, when different compounds were evaluated for differences in diffusion through stratum corneum, they all had very similar diffusion coefficients when anatomical site was controlled for (Rougier et al., 1990).

It is important to understand the proteins involved in the stratum corneum, which are collectively contributing to this strong barrier. Stratum corneum is made of corneocytes, which are keratin threads weaved together in an organized fashion, holding a large amount of water amongst them. There are about 12–16 layers of these corneocytes, each layer with an average thickness of 1 μm. Variations are seen based on anatomical site and UV exposure (Nemes, Steinert, 1999). Crystalline lamellar lipid regions envelop around corneocytes. These lamellar bodies are generated by keratinocytes in the underlying stratum granulosum and stratum spinosum. Maturation pushes these lamellar bodies higher into the stratum corneum, where they are simultaneously degraded, causing release of ceramides and free fatty acids. The release of these substances results in their fusion, which forms the aforementioned lamellar lipid bilayer (Norlén, 2006; Proksch et al., 2008). In addition to corneocytes being surrounded by this lamellar lipid bilayer, specific proteins, namely, loricrin and involucrin, form a tight envelope around them. Attached to them are ceramides that hold onto water. These proteins and lipids simply lay the groundwork of the skin’s barrier. Other proteins, including Natural Moisturizing Factor and filaggrin, amongst many others, further trap water and contribute to the meshwork of the stratum corneum (Hui et al., 2013). Note that permeation through the stratum corneum not only depends on the proteins that make up its core, but also depends on the compound’s solubility, pH, concentration, and partitioning between the stratum corneum and the vehicle.

### Complexity of *in vivo* binding

The further research that is performed, the further we learn about the sheer complexity of the human skin, much of which has yet to be clarified. Law et al.’s recent review is considered up-to-date on the factors affecting percutaneous absorption of a compound in humans. Table 4 details twenty pertinent factors that have been studied up to this point. Law et al. summarizes all the relevant factors, a few of which will be detailed here. First, the compound being applied to the skin has its own chemical properties that affect its absorption. Smaller molecules are more permeable, lipophilic drugs are more easily absorbed due to the lipophilic nature of the stratum corneum (although highly lipophilic drugs are not as easily absorbed), and basic drugs have an easier time penetrating the skin as well. The reason for this is that the skin’s proteins are slightly basic and unionized forms of drugs pass through membranes. Basic drugs are more likely to be un-ionized, thus, it follows that basic drugs are more readily absorbed. It is also important to discuss the partition coefficient of a drug, which is the relative concentration ratio of the compound between two immiscible solvents. It can help us infer the solubility differences of the compound between the two phases. It is important to focus on the octanol:water partition coefficient (also known as oil to water coefficient) and the formulation:skin partition coefficient (also known as the cream or ointment relative to the stratum corneum). Hydrophobic compounds have a high octanol:water partition coefficient and are more likely to be found near lipid bilayers. On the other hand, hydrophilic compounds have a low octanol:water partition coefficient and are more likely to be found in aqueous areas. The formulation:skin partition coefficient is based on the actual formulation of the drug (Law et al., 2020).

The vehicle used in the compound plays a role in the latters absorption. More occlusive vehicles aid in absorption, whereas water-based vehicles do not aid all types of drug absorption, but do help increase absorption of hydrophilic drugs. An example of an occlusive vehicle is betamethasone dipropionate 0.1% ointment being used as opposed to a cream—this change to an oil-based vehicle helps increase permeability (Law et al., 2020).

Application frequencies, sites of application, and population variability are also important factors to consider. Application frequency is an area of minimal research, however, with corticosteroids a high concentration single dose may have a greater impact on absorption than multiple doses of a lower concentration (Wester et al., 1977). Other factors that are vital to consider include skin hydration and skin health/integrity.

As previously discussed, skin compartments and protein binding play a role in permeation of a compound. An important term to know here is “substantivity”, which can be seen as the power of the drug to remain in the stratum corneum. When undergoing topical formulation, it is important to ask ourselves that during activities such as sweating, exercise or water exposure—does the compound remain effective? And for how long? Thus, the higher the substantivity, the greater chance that the drug has a prolonged effect both topically and systemically (Law et al., 2020).

This leads to our discussion of systemic absorption. There are three major factors to consider here—substantivity, binding affinity to skin substances, and binding activity to blood components. Substantivity, as previously discussed, is the concept of if the drug remains in the stratum corneum and/or other skin compartments, potentially increasing its duration of action. Binding affinity to skin substances includes proteins, lipids, hair follicles, and sweat glands. As chemicals descend down the skin, and enter the dermis, they encounter vascular blood supply. Affinity for blood substances such as albumins and plasma proteins is important to take into account here. Moreover, some drugs have vasodilatory properties, increasing their systemic effect (Law et al., 2020).

Other environmental factors such as exfoliation, washing, massaging, and skin to skin contact are included in the complexity of percutaneous absorption. Furthermore, metabolic factors, discussed previously, play an important role in the effect of the drug. Aging skin can also pose its own complexities, which may affect the absorption of a drug. The only *in vivo* study that evaluated this was done in 1989 by Roskos et al., who evaluated the effects of testosterone, estradiol, hydrocortisone, benzoic acid, acetylsalicylic acid, and caffeine through urinary excretion profiles. Their results showed a significantly lower permeation of hydrocortisone, benzoic acid, acetylsalicylic acid, and caffeine in older subjects (greater than 65 years old, compared to the 18–40 years old group). Testosterone and estradiol had similar systemic uptake in both age groups. They concluded that aging skin has a relatively lower lipid content which made substances with lower lipid solubility (e.g., hydrocortisone) more susceptible to permeation changes (Roskos et al., 1989).

Finally, in addition to the twenty factors listed in Table 4, it is important to note the effect of drugs on damaged skin, particularly in the realm of occupational skin hazards. Gattu’s review showed different effects of compounds on diseased skin—some had an increase in penetration through damaged skin, while others had an equal or decreased penetration. Their damaged skin results showed that hydrophilic compounds generally show higher penetration than lipophilic compounds. This can be explained by patterns already observed in healthy skin. As a general rule, lipophilic compounds are more absorbed through the skin’s lipid bilayer, while hydrophilic compounds have a more difficult time being absorbed. Thus, the absorption of hydrophilic compounds may be more reliant on damage to epidermis (Gattu and Maibach, 2011). Others postulate that severity of disease plays an important role in percutaneous absorption. There is still much to be learned in the area of damaged skin (Turpeinen, 1988).

### Validation of *in vitro* studies

We are aware of the difficulty of obtaining human skin. Human and animal studies also introduce ethical concerns. The goal is to extrapolate data from *in vitro* and *ex vivo* studies for the approval of compounds to be used on human skin for a variety of dermatologic concerns. Lehman et al. compared several *in vitro* and *in vivo* studies in order to evaluate their similarity. Figure 3 graphically depicts their results. They split their data sets into all data (Figure 3A) and harmonized data (Figure 3B), with harmonized data being more trustworthy as several confounding variables were accounted for such as anatomical site, drug concentration, vehicle used, and experiment length, amongst others. Ratios were created between *in vitro* and *in vivo* studies, referred to as the IVIV ratio, which would ideally be a value close to or equal to 1.0. Amongst all 92 data sets, the mean IVIV ratio was 1.6. There were anomalies that had close to a 20-fold difference between *in vitro* and *in vivo* studies, but 85% of data sets had a difference of less than 3-fold. Once data was harmonized, which excluded 81 of the datasets, the IVIV ratio was 0.96 with less than a 2-fold difference between all *in vitro* vs. *in vivo* studies. Conclusively, using 11 datasets that were harmonized and matched to minimize confounding factors, we see an almost perfect 1:1 ratio between evidence obtained from *in vitro* to *in vivo* studies (Lehman et al., 2011). Although prospective studies must be done, this is an encouraging start.

## Conclusion

Skin is a unique organ; the fascinating barrier that the stratum corneum provides not only selectively allows certain penetrants to be absorbed, but also helps control homeostasis in the body by controlling the amount of water that can exit the body. Since the 1960s, advances have been made in the field of dermatology—compounds are evaluated through *in vitro*, *in vivo*, and *ex vivo* methods. Now, due to ethical and financial implications, as well as difficulty obtaining samples, human and animal samples are not as readily utilized to study the penetration of compounds across the skin. *In vitro* and *ex vivo* studies have become more utilized due to this. When extraneous factors are controlled for, the results obtained from *in vitro* studies can be utilized to make conclusions for use on human *in vivo* skin. Although there is still much to be learned on the complexity of skin, current research provides for a promising future of percutaneous absorption in dermatology.

Taken together, Burch and Windsor, and Tregear—three-quarters of a century ago opened the discussion - and as summarized here, *in vitro* penetration studies have become important tools for dermatopharmacology and dermatotoxicology insights. Although several standardized protocols exist (FDA, EPA, EMA), it is likely that as more experimental data becomes available, further protocol changes will occur. With incoming data, the findings of Lehman et al. (Lehman et al., 2011) will likely lead to closer in vitro-in vivo correlations. Law summarizes 20 steps identified for human *in vivo* penetration (Law et al., 2020)—and has since identified 4 further steps (in preparation). Taking these steps into consideration when interpreting *in vitro* data, should lead to closer in vitro-in vivo correlation. The data should add to the veracity of the assay, not only for bioequivalence evaluation, but as an aid in formulation development and quality control in manufacturing. (Schaefer and Redelmeier, 1996), (Bronaugh, 1985), (Menczel and Maibach, 1970), (Maibach, 2023)

## Footnotes

† This publication is modified from Dr. Howard Maibach’s keynote address at the FDA’s IVPT/IVRT virtual workshop on August 18–20, 2021. Web: https://complexgenerics.org/media/SOP/complexgenerics/pdf/Conference-Slides/D1-1%20Howard%20Maibach%20IVPT%20 PERMEATION%20TEST%20-%20FDA%20VIRTUAL.pdf

## In memoriam

This manuscript is dedicated to the memory of Dr. Ken Wallers and Dr. Keith Brain—long-time supporters of percutaneous penetration research and education.
